# Economic burden of secondary hyperparathyroidism in Germany: a matched comparison

**DOI:** 10.1007/s11255-022-03425-9

**Published:** 2022-12-08

**Authors:** Helmut Reichel, Eric Seibert, Frank-Peter Tillmann, Isabella Barck, Astride Grava, Kim Maren Schneider, Dominic Meise

**Affiliations:** 1 Scientific Institute for Nephrology, Düsseldorf, Germany; 2 Nephrological Center Villingen-Schwenningen, Villingen-Schwenningen, Germany; 3 Department of Internal Medicine II, Martin Luther University Halle-Wittenberg, Halle (Saale), Germany; 4 Department of Medicine I-Nephrology, Transplantation & Medical Intensive Care, Medical Center Cologne-Merheim, University Witten/Herdecke, Cologne, Germany; 5 Nephrological Centre Ibbenbüren, Ibbenbüren, Germany; 6 Vifor Pharma Deutschland GmbH, Munich, Germany; 7 Vifor Pharma Management AG, Glattbrugg, Switzerland; 8 Xcenda GmbH, Hannover, Germany

**Keywords:** Secondary hyperparathyroidism, Chronic kidney disease, Cost analysis, Burden of illness, Germany, Claims data

## Abstract

**Purpose:**

Secondary hyperparathyroidism (SHPT) of renal origin is a progressive complication in chronic kidney disease (CKD) and is associated with serious osseous and non-osseous complications, CKD progression, and economic burden for healthcare systems worldwide. We aimed at assessing characteristics, healthcare resource utilization, and costs of incident SHPT patients in CKD stage 3 (CKD3) and 4 (CKD4), using administrative claims data.

**Methods:**

German claims data were used to identify CKD3 and CKD4 patients, who were stratified by the occurrence of incident SHPT. Patients with SHPT were matched 1:1 to non-SHPT patients with the same CKD stage using propensity scores. Matched groups were compared during a 2-year follow-up period.

**Results:**

Overall, 1156 CKD3 and 517 CKD4 incident SHPT patients and their respective matches were identified. Mean number of all-cause hospitalizations were significantly higher among SHPT patients (2.7 vs. 2.0 in CKD3, 2.8 vs. 1.5 in CKD4) during follow-up. Similarly, the mean number of outpatient encounters was significantly higher among the SHPT cohorts (95.0 vs. 64.3 in CKD3, 101.4 vs. 49.8 in CKD4). SHPT patients progressed to CKD5 more often (6.1% vs. 1.2% from CKD3, 26.7% vs. 2.9% from CKD4, both *P* < 0.01) resulting in a higher proportion of dialysis (6.1% vs. 1.3% in CKD3, 22.1% vs. 3.7% in CKD4, both *P* < 0.01). Consequently, average all-cause healthcare costs significantly increased per patient (€19,477 vs. €15,115 in CKD3, €25,921 vs. €12,265 in CKD4).

**Conclusions:**

Patients with CKD3&4 and incident SHPT of renal origin presented with significantly higher healthcare resource utilization and costs, as well as increased disease progression compared to non-SHPT patients.

## Introduction

Chronic Kidney Disease (CKD) is a global health burden associated with increased risk of cardiovascular morbidity, mortality, reduced health related quality of life, and a substantial economic cost burden to health systems worldwide [1]. While the age standardized global prevalence of CKD remained stable between 1990 and 2017, the all-age prevalence increased by 29.3% [2] and the burden of CKD has risen considerably in recent years [3].

A common complication among CKD patients is secondary hyperparathyroidism (SHPT), a complex alteration in bone and mineral metabolism characterized by excessive parathyroid hormone (PTH) production and parathyroid gland hyperplasia [4–6]. SHPT occurs in 40–80% of CKD patients in stage 3 and 4, respectively [7, 8].

Poorly managed SHPT may cause metabolic bone disease and is associated with further complications such as cardiovascular disease (CVD) as well as cardiovascular-associated and all-cause mortality [4, 9–11]. Furthermore, the associated healthcare costs of SHPT and related clinical events are high and place an economic burden on health systems [12].

To the best of our knowledge, published real-world data on the burden and excess costs of SHPT in Germany from the perspective of the statutory health insurance system (SHI) is lacking. Therefore, the aim of this study was to assess the clinical characteristics, as well as excess healthcare resource utilization, and healthcare costs of German patients with incident SHPT of renal origin in CKD stage 3 (CKD3) and stage 4 (CKD4), using administrative SHI claims data.

## Methods

### Study design and data source

This retrospective, matched cohort analysis used administrative claims data from the “Institut für angewandte Gesundheitsforschung Berlin” [Institute for Applied Health Research Berlin] (InGef) research database in Germany. The database includes anonymized claims data of approximately 4 million individuals and is in line with German data protection regulations. The research sample is adjusted in terms of age and gender to represent the German population and has been proven to be in good accordance with respect to morbidity, mortality as well as drug usage compared to the general population in Germany [13].

Claims data from the participating SHI funds are joined in a specialized trust center, anonymized, and subsequently transferred to InGef. As the raw dataset is not allowed to leave the secured storage facilities, all analyses were conducted by an InGef analyst in accordance with a pre-specified study protocol.

The database provides information on a range of different healthcare sectors such as the inpatient (hospital) setting, outpatient physicians, and the pharmacy sector. Detailed information on the resource use and cost of provided services can be analyzed [13], with inherent limitations, such as diagnoses in the outpatient setting are only available on a quarterly basis.

As claims data are recorded for accounting purposes, not for clinical research, and no electronic medical records or other clinical parameters were used, no ethical approval or consent from an ethics committee or review board was required for this study.

### Study population and cohorts

Overall, the patient selection process aimed to identify patients with CKD3 and CKD4 and to stratify these patients by the occurrence of incident SHPT of renal origin within the enrollment period between January 1st, 2015 and March 31st, 2017. The total study period including pre- and post-index periods spanned from January 1st, 2014 to December 31st, 2018.

Patients with CKD stage 3 and 4 were identified in the inpatient or outpatient setting using International Statistical Classification of Diseases and Related Health Problems, 10th revision, German Modification (ICD-10-GM) codes N18.3 [chronic kidney disease, stage 3] and N18.4 [chronic kidney disease, stage 4]. In case the ICD-10-GM code for CKD3 or CKD4 was recorded in the outpatient sector, further validation was required by a second inpatient or outpatient ICD-10-GM code for CKD of any severity within a year’s (± 3 quarters) range of the first CKD diagnosis.

The identified CKD3 and CKD4 patients were stratified by the occurrence of SHPT using either an ICD-10-GM code N25.8 [other disorders resulting from impaired renal tubular function, including secondary hyperparathyroidism of renal origin] in the inpatient or outpatient setting, or a prescription of paricalcitol, alfacalcidol or calcitriol in the outpatient setting, which are commonly administered by nephrologists for the treatment of SHPT of renal origin. Outpatient diagnoses or prescriptions had to be validated by at least one additional outpatient diagnosis or prescription within a year’s (± 3 quarters) range, allowing any combination of outpatient diagnosis and prescription (e.g., two outpatient diagnoses or two prescriptions). Patients were excluded per protocol from the non-SHPT cohorts if they had any SHPT diagnosis (also of non-renal origin) or prescription for paricalcitol, alfacalcidol or calcitriol during the entire study period.

For patients with SHPT, the first ICD-10-GM code or prescription indicating SHPT marked the index event and index quarter. The highest CKD stage (3 or 4) within the index quarter marked the index CKD stage for the SHPT patients. To identify only incident SHPT, patients were excluded if they presented with any diagnosis or prescription for SHPT as described above, within a year before the index quarter. For the CKD3 and CKD4 patients without a diagnosis or prescription for SHPT, a random diagnosis for CKD3 or CKD4 was chosen as the index event and index CKD stage.

Patients from all cohorts were excluded, if they were below the age of 18 at index, or if they presented with any evidence for dialysis treatment or kidney transplant in the year before index. Furthermore, patients with CKD stage decline, e.g., from stage 4 to stage 2 were excluded.

The patient selection process did not aim to create evidence on the epidemiology (incidence/prevalence) of SHPT but was intended to create a study population to assess the economic burden of the condition.

### Matching

Each patient in the SHPT cohort with CKD3 (CKD4) was matched to one control patient from the CKD3 (CKD4) cohort without SHPT via propensity score matching. The propensity scores were calculated using logistic regression, with regressors age, gender, updated Charlson Comorbidity Score (CCI) [14], index quarter, New York Heart Association (NYHA) class for heart failure (assessed via ICD-10-GM codes), and whether the index CKD stage was determined in the hospital to account for disease severity.

Matching fit was assessed using standardized mean differences (SMD), with a SMD below 0.1 indicating acceptable balance between the cohorts [15].

### Outcomes

Outcomes were analyzed in the index quarter (demographic characteristics) or in a follow-up period of up to 2 years (including the index quarter). Specific comorbidities of interest such as cardiovascular disorders and SHPT-related complications, costs, outpatient visits, and hospitalizations were also analyzed in the 1-year (4 quarters) period before index (baseline), to further assess whether the matched cohorts were comparable before their respective index events.

Mortality was analyzed during follow-up. All-cause healthcare resource utilization was assessed in terms of hospitalizations, length of hospital stays, and outpatient visits. CVD-related healthcare resource utilization was assessed in terms of hospitalizations with an ICD-10-GM code (primary or secondary) for myocardial infarction, heart failure or stroke. Progression to stage 5 CKD was assessed using ICD-10-GM codes, while initiation of dialysis was identified using ICD-10-GM, operation and procedure codes, or EBM codes [German remuneration scheme for outpatient care]. All-cause healthcare costs in total and stratified by healthcare sector (inpatient care, outpatient care, pharmaceuticals, devices, remedies, and aids) were analyzed at 12- and 24-month follow-up. All continuous outcomes were compared using bootstrapping *t*-tests while chi^2^ tests or Fisher's exact test (for *n* < 5) were applied for dichotomous variables. A *P*-value of < 0.05 was considered as statistically significant.

## Results

### Population characteristics

After applying all study criteria, 1158 incident SHPT CKD3 and 521 incident SHPT CKD4 patients as well as 64,962 CKD3 and 6543 CKD4 patients without evidence of SHPT were identified. Two additional patients in the incident SHPT CKD3 cohort and four patients in the incident SHPT CKD4 cohort were excluded after the 1:1 propensity score matching, as no suiting matching partner was available in the non-SHPT cohorts. Overall, good balance with respect to all matching variables was achieved, as all SMDs remained below 0.1 (Table 1). The mean age of the CKD3 groups was 74.4 years in the SHPT and 74.8 years in the non-SHPT cohort. The CKD4 groups were slightly older, with a mean age of 76.9 years in the SHPT and 77.9 years in the non-SHPT cohort. The proportion of male patients with CKD3 was higher in both the SHPT and the non-SHPT cohort (56.6% and 57.4%). In contrast, there were more female patients with CKD4, 53.0% in the SHPT and 55.3% in the non-SHPT cohort. More than 35% of patients in all cohorts had at least one diagnosis code for heart failure, whereas for a high proportion the NYHA class was not specified (unspecific diagnosis) (Table 1). Index quarters between the cohorts were balanced (data not shown).

**Table 1:** Patient characteristics after matching

Parameter	CKD3 SHPT	CKD3 no SHPT	SMD	CKD4 SHPT	CKD4 no SHPT	SMD
Number of patients, *n* (%)	1156 (100.0)	1156 (100.0)		517 (100.0)	517 (100.0)	
Age in years, mean (SD)	74.4 (10.2)	74.8 (10.8)	– 0.03	76.9 (10.3)	77.9 (11.6)	– 0.09
Gender, *n* (%)						
Male	654 (56.6)	663 (57.4)	– 0.02	243 (47.0)	231 (44.7)	0.05
Female	502 (43.4)	493 (42.6)	0.02	274 (53.0)	286 (55.3)	– 0.05
CCI, mean (SD)	3.6 (2.4)	3.6 (2.4)	0.00	3.8 (2.4)	3.8 (2.5)	– 0.01
CKD hospitalization in the index quarter, *n* (%)						
No	855 (74.0)	847 (73.3)	0.02	301 (58.2)	320 (61.9)	– 0.08
Yes	301 (26.0)	309 (26.7)	– 0.02	216 (41.8)	197 (38.1)	0.08
NYHA class, *n* (%)						
No NYHA class	724 (62.6)	750 (64.9)	– 0.05	308 (59.6)	308 (59.6)	0.00
NYHA 1	16 (1.4)	11 (1.0)	0.04	9 (1.7)	13 (2.5)	– 0.05
NYHA 2	69 (6.0)	68 (5.9)	0.00	24 (4.6)	17 (3.3)	0.07
NYHA 3	114 (9.9)	105 (9.1)	0.03	41 (7.9)	41 (7.9)	0.00
NYHA 4	59 (5.1)	54 (4.7)	0.02	45 (8.7)	45 (8.7)	0.00
NYHA unknown	174 (15.1)	168 (14.5)	0.01	90 (17.4)	93 (18.0)	– 0.02

*CCI (updated)* Charlson Comorbidity Index, *CKD* chronic kidney disease, *NYHA* New York Heart Association, *SD* standard deviation, *SMD* standardized mean difference, *SHPT* secondary hyperparathyroidism

### All-cause mortality and comorbidities

During the 1-year pre-index period, patients were comparable with respect to most comorbidities of interest, although there was a statistically significantly higher proportion of patients with chronic ischemic heart disease and calcium metabolism disorders in the SHPT CKD3 group.

Comorbidities of interest that were statistically significantly more prevalent in SHPT CKD3 patients in comparison to their matched non-SHPT counterparts during the 24-month post-index period were vitamin D deficiency, chronic ischemic heart disease, congestive heart failure, osteoporosis, renal osteodystrophy, and calcium metabolism disorders. A composite indicator for having any acute myocardial infarction, recurrent myocardial infarction, chronic ischemic heart disease, congestive heart failure or atherosclerosis was also significantly more prevalent in the SHPT CKD3 cohort compared to their non-SHPT controls. Among SHPT CKD4 patients, the prevalence of vitamin D deficiency, acute myocardial infarction, congestive heart failure, renal osteodystrophy, disorders of phosphate metabolism, and disorders of calcium metabolism were significantly increased compared to the matched cohort of non-SHPT patients (Table 2).

**Table 2:** Specific comorbidities during the 1-year baseline period and 2-year follow-up

Comorbidity	CKD3 SHPT	CKD3 no SHPT	*P*-value	CKD4 SHPT	CKD4 no SHPT	*P*-value
Number of patients, *n* (%)	1156 (100.0)	1156 (100.0)	–	517 (100.0)	517 (100.0)	–
1-year baseline period:						
Vitamin D deficiency (E55.9), *n* (%)	95 (8.2)	73 (6.3)	0.08	39 (7.5)	24 (4.6)	0.05
Acute myocardial infarction (I21), *n* (%)	74 (6.4)	58 (5.0)	0.15	37 (7.2)	31 (6.0)	0.45
Recurrent myocardial infarction (I22), *n* (%)	< 5	0	–	< 5	< 5	–
Chronic ischemic heart disease (I25), *n* (%)	519 (44.9)	454 (39.3)	< 0.01	198 (38.3)	193 (37.3)	0.75
Congestive heart failure (I50), *n* (%)	432 (37.4)	406 (35.1)	0.26	209 (40.4)	209 (40.4)	1.00
Atherosclerosis (I70), *n* (%)	263 (22.8)	268 (23.2)	0.80	95 (18.4)	112 (21.7)	0.19
Any CVD (I21, I22, I25, I50, or I70), *n* (%)	732 (63.3)	704 (60.9)	0.23	318 (61.5)	318 (61.5)	1.00
Osteoporosis (M80, M81), *n* (%)	154 (13.3)	159 (13.8)	0.76	66 (12.8)	79 (15.3)	0.24
Renal osteodystrophy (N25.0), *n* (%)	< 5	0	–	< 5	0	–
Disorders of phosphate metabolism (E83.38, E83.39), *n* (%)	< 5	< 5	–	< 5	< 5	–
Calcium metabolism disorders (E83.5), *n* (%)	30 (2.6)	10 (0.9)	< 0.01	17 (3.3)	10 (1.9)	0.17
2-year follow-up period:						
Vitamin D deficiency (E55.9), *n* (%)	464 (40.1)	113 (9.8)	< 0.01	187 (36.2)	30 (5.8)	< 0.01
Acute myocardial infarction (I21), *n* (%)	114 (9.9)	94 (8.1)	0.15	61 (11.8)	38 (7.4)	< 0.05
Recurrent myocardial infarction (I22), *n* (%)	0 (0.0)	< 5	–	< 5	< 5	–
Chronic ischemic heart disease (I25), *n* (%)	592 (51.2)	520 (45.0)	< 0.01	240 (46.4)	212 (41.0)	0.08
Congestive heart failure (I50), *n* (%)	573 (49.6)	485 (42.0)	< 0.01	306 (59.2)	261 (50.5)	< 0.01
Atherosclerosis (I70), *n* (%)	357 (30.9)	336 (29.1)	0.34	146 (28.2)	126 (24.4)	0.16
Any CVD (I21, I22, I25, I50, or I70), *n* (%)	842 (72.8)	782 (67.7)	< 0.01	382 (73.9)	364 (70.4)	0.21
Osteoporosis (M80, M81), *n* (%)	241 (20.9)	186 (16.1)	< 0.01	94 (18.2)	95 (18.4)	0.94
Renal osteodystrophy (N25.0), *n* (%)	17 (1.5)	< 5	< 0.01	10 (1.9)	0	< 0.01
Disorders of phosphate metabolism (E83.38, E83.39), *n* (%)	11 (1.0)	< 5	0.06	21 (4.1)	< 5	< 0.01
Calcium metabolism disorders (E83.5), *n* (%)	84 (7.3)	28 (2.4)	< 0.01	40 (7.7)	17 (3.3)	< 0.01

Patient counts below 5 are reported as < 5 due to data protection regulations. Used ICD-10-GM codes are reported in parentheses

*CVD* cardiovascular disease, *CKD* chronic kidney disease, *SHPT* secondary hyperparathyroidism

All-cause mortality was significantly higher among non-SHPT CKD3 patients in stage 3 (20.6% vs. 16.2%, *P* < 0.01) and stage 4 (43.3% vs. 35.4%, *P* < 0.01) in comparison to the SHPT counterparts. This led to a 5.9% increase in follow-up time among SHPT CKD3, and 17.9% increased follow-up time among SHPT CKD4 patients (Table 3).

**Table 3:** Mortality and length of follow-up

Parameter	CKD3 SHPT	CKD3 no SHPT	*P*-value	CKD4 SHPT	CKD4 no SHPT	*P*-value
Number of patients, *n* (%)	1156 (100.0)	1156 (100.0)	–	517 (100.0)	517 (100.0)	–
Death during follow-up, *n* (%)	187 (16.2)	238 (20.6)	< 0.01	183 (35.4)	224 (43.3)	< 0.01
Length of follow-up in days, mean (SD)	671.8 (157.9)	634.5 (210.6)	< 0.01	574.1 (245.6)	487.0 (303.2)	< 0.01

*CKD* chronic kidney disease, *SD* standard deviation, *SHPT* secondary hyperparathyroidism

### Healthcare resource utilization

In the year before index, the SHPT patients were comparable to their respective control groups with respect to the proportion of hospitalized patients with slightly higher proportions in the SHPT cohorts (CKD3: 50.6% vs. 47.4%; *P* = 0.12; CKD4: 52.8% vs. 48.6%; *P* = 0.17, not shown in tables).

During the individual 2-year follow-up period, the proportion of patients with at least one hospitalization among CKD3 patients was 74.2% in the SHPT cohort and 67.0% in the control group (*P* < 0.01), while 78.7% of SHPT patients with CKD4 were hospitalized compared to 71.0% of their matched counterparts (*P* < 0.01). This translated to a higher average rate of hospitalizations among the SHPT cohorts (2.7 vs. 2.0 for CKD3; 2.8 vs. 1.5 for CKD4) and the average length of hospital stay was higher in the SHPT cohorts (26.9 days vs. 20.4 days for CKD3 patients; 34.7 days vs. 16.7 days for CKD4 patients). All of the described differences with respect to hospitalizations were statistically significant (*P* < 0.01). Additionally, the proportion of patients with a CVD-related hospitalization for myocardial infarction, heart failure or stroke was significantly higher among SHPT patients with CKD3 (37.1% vs. 30.4%; *P* < 0.01) and CKD4 (46.4% vs. 36.2%; *P* < 0.01) (Table 4).

**Table 4:** Healthcare resource utilization during 2-year follow-up

Healthcare resource utilization	CKD3 SHPT	CKD3 no SHPT	*P*-value	CKD4 SHPT	CKD4 no SHPT	*P*-value
Number of patients, *n* (%)	1156 (100.0)	1156 (100.0)	–	517 (100.0)	517 (100.0)	–
All-cause outpatient visits, mean (SD)	95.0 (55.4)	64.3 (43.5)	< 0.01	101.4 (93.7)	49.8 (41.3)	< 0.01
Outpatient nephrologist care, *n* (%)	878 (76.0)	224 (19.4)	< 0.01	384 (74.3)	100 (19.3)	< 0.01
All-cause hospitalizations						
Number of patients with hospitalization, *n* (%)	858 (74.2)	774 (67.0)	< 0.01	407 (78.7)	367 (71.0)	< 0.01
Number of hospitalizations, mean (SD)	2.7 (3.2)	2.0 (3.0)	< 0.01	2.8 (3.2)	1.5 (1.8)	< 0.01
Length of hospital stay in days, mean (SD)	26.9 (44.6)	20.4 (39.7)	< 0.01	34.7 (58.9)	16.7 (34.1)	< 0.01
CVD-related hospitalizations						
Number of patients with CVD-related hospitalization, *n* (%)	429 (37.1)	351 (30.4)	< 0.01	240 (46.4)	187 (36.2)	< 0.01
Number of CVD-related hospitalizations, mean (SD)	0.8 (1.5)	0.6 (1.1)	< 0.01	1.1 (1.8)	0.5 (0.8)	< 0.01
Length of CVD-related hospital stay in days, mean (SD)	11.0 (24.4)	7.7 (21.3)	< 0.01	16.7 (31.2)	6.7 (13.6)	< 0.01

*CVD* cardiovascular disease, *CKD* chronic kidney disease, *SD* standard deviation, *SHPT* secondary hyperparathyroidism

In the year before index, the average number of all-cause outpatient visits among SHPT CKD3 patients was 41.4, compared to 36.5 in the control group (*P* < 0.01), while the number was comparable between SHPT CKD4 patients and their matched counterparts (39.5 vs. 38.1; *P* = 0.36, not shown in tables). In the follow-up period, mean average all-cause outpatient visits significantly differed between SHPT and control patients in CKD3 (95.0 vs. 64.3; *P* < 0.01) and CKD4 (101.4 vs. 49.8; *P* < 0.01). The proportion of patients with at least one outpatient visit at a nephrologist during the baseline period was roughly twice as high in the SHPT CKD3 (28.4% vs. 14.6%; *P* < 0.01) and SHPT CKD4 groups (31.3% vs. 14.51%; *P* < 0.01). This difference increased further within the post-index period to 76.0% vs 19.4% for CKD3 patients and to 74.3% vs. 19.3% for CKD4 patients (Table 4).

The proportion of patients progressing to CKD stage 5 was 6.1% for SHPT CKD3 patients and 1.2% for their controls (*P* < 0.01), as well as 26.7% for SHPT CKD4 patients compared to 2.9% for CKD4 only patients (*P* < 0.01). Consequently, the initiation of dialysis was observed substantially more often in the SHPT cohorts (Table 5).

**Table 5:** Progression to stage 5 and dialysis during 2-year follow-up

Progression	CKD3 SHPT	CKD3 no SHPT	*P*-value	CKD4 SHPT	CKD4 no SHPT	*P*-value
Number of patients, *n* (%)	1156	1156	–	517	517	–
Progression to CKD5, *n* (%)	71 (6.1)	14 (1.2)	< 0.01	138 (26.7)	15 (2.9)	< 0.01
Dialysis initiation, *n* (%)	71 (6.1)	15 (1.3)	< 0.01	114 (22.1)	19 (3.7)	< 0.01

*CKD* chronic kidney disease, *SHPT* secondary hyperparathyroidism

### All-cause healthcare costs

In the 1-year pre-index period, there was no statistically significant difference observable for the total all-cause costs between the groups, although patients who developed SHPT had higher costs on average (average costs + 15.4% in CKD3 patients (*P* = 0.07) and + 7.2% (*P* = 0.44) in CKD4 patients with SHPT, not shown in tables).

**Fig. 1:**
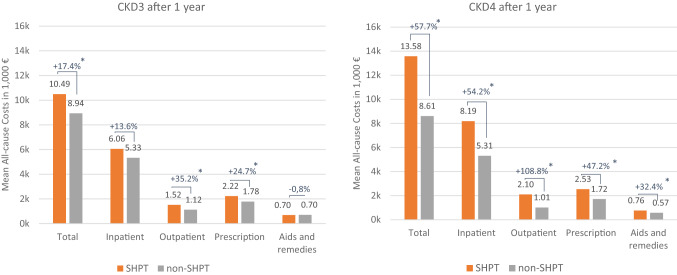
Healthcare costs after 1 year

Total all-cause healthcare costs among the cohorts after 12 months of follow-up were significantly higher (17.4% for CKD3 and 57.7% for CKD4; *P* < 0.01) for both SHPT cohorts (Fig. 1), with the absolute incremental differences being €1554 (CKD3) and €4970 (CKD4), respectively. The SHPT cohorts had higher healthcare costs in all observed healthcare sectors, with the exception for aids and remedies among CKD3 patients, where the costs were comparable between SHPT and non-SHPT patients. The main cost driver for all cohorts was the inpatient sector that accounted for more than half of total healthcare costs after 12 months.

**Fig. 2:**
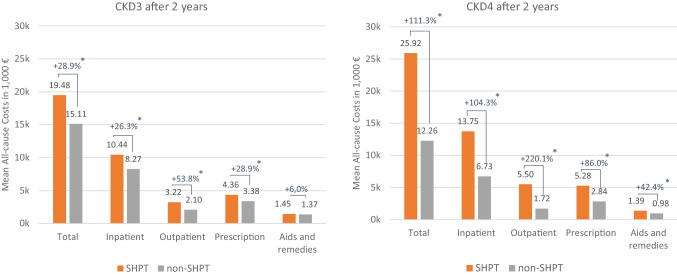
Healthcare costs after 2 years

When considering the up to 24 months post-index period, SHPT CKD3 patients had 28.9% increased all-cause healthcare costs and SHPT CKD4 patients had 111.3% increased healthcare costs compared to their matched controls (both *P* < 0.01), with incremental cost differences of €4362 (CKD3) and €13,656 (CKD4), respectively (Fig. 2). Costs in all healthcare sectors were substantially higher in the SHPT cohorts, except for aids and remedies costs among CKD3 patients.

## Discussion

To the best of our knowledge, this retrospective analysis of a large German claims database is the first to assess the excess burden associated with SHPT in CKD patients within the German healthcare system. As the underlying database can be considered nationally representative in terms of demographic and clinical characteristics [13], the results of this study can be referred to as a real-world representation of the German population in general. Furthermore, it adds to the literature as we assessed a specific sample of SHPT and non-SHPT patients stratified by pre-dialysis CKD stages 3 and 4 not reported elsewhere.

The patient selection process revealed that in a real-world setting, only a small proportion of CKD3 and CKD4 patients have a recorded diagnosis for SHPT, which is contrary to the findings of studies utilizing laboratory parameters, where 40–80% of CKD3/CKD4 patients are suffering from SHPT [7]. SHPT has been described as underdiagnosed and undertreated [16], a finding that is underlined by our results. Given the observed differences between the SHPT and the non-SHPT groups in our study, this would mean that with high probability we underestimated the actual disease burden for SHPT patients. Current clinical practice guidelines for the diagnosis, evaluation, prevention, and treatment of chronic kidney disease-mineral and bone disorders state that the PTH target range in non-dialysis CKD patients is not known [17]. This likely results in unclear SHPT diagnosis criteria in clinical practice and hence, possibly undercoding.

The average age of 74–78 years within the groups of interest were comparable to another German study of CKD3 and 4 patients utilizing data from nephrological clinics where the average age was 75 [18].

In most cases, the prevalence of selected cardiovascular and other comorbidities such as congestive heart failure, vitamin D deficiency, and osteoporosis was significantly higher in the SHPT cohorts, possibly leading to higher resource use. This finding was expected with regard to the known associations between SHPT with bone and cardiovascular diseases [19]. However, some of these differences were also present in the baseline period, as for example chronic ischemic heart disease and calcium metabolism disorders were more prevalent among CKD3 SHPT patients. All-cause mortality was higher among non-SHPT patients in the CKD3 and CKD4 cohorts. This finding contrasts with the fact that SHPT is generally associated with higher all-cause and cardiovascular mortality. For example, a retrospective study by Kovesdy et al. has shown that raised PTH levels were associated with an increased all-cause mortality among male pre-dialysis CKD patients in the US [20]. Our finding is especially surprising, as the patient cohorts were matched with respect to several indicators for health status such as CCI or NYHA class. Therefore, death in these patients might possibly be related to other factors such as comorbidities developed after matching or not included in the set of those variables that were used for the matching. Furthermore, malnutrition has been shown to be associated with lower PTH levels and higher mortality among hemodialysis patients [21], but cannot be depicted with claims data to a sufficient extent. A possible explanation for the difference in mortality in our investigation is the increased medical attention to the SHPT patients shown by e.g., more (nephrologist-) outpatient visits and hence monitoring, which in turn might result in the lower mortality. This effect has been demonstrated in several studies for CKD patients [22, 23]. For example, Liu et al. [22] investigated the effect of outpatient nephrology consultations in pre-dialysis CKD4 patients. In their assessment of laboratory and administrative data from Canada, they found that patients with nephrologist visits had a 12% lower mortality compared to patients without a nephrologist consultation.

The main finding of our study is that diagnosed SHPT patients in CKD3 and CKD4 had significantly increased healthcare resource use, healthcare costs, as well as progression to CKD5 and dialysis when compared to a matched sample of patients within the same CKD stage and no SHPT. Specifically, we observed more patients with hospitalizations, as well as differences in hospitalizations rates of more than 30.6% in CKD3 patients and 86.6% in CKD4 patients with SHPT compared to their peers. Healthcare costs after the up to 2-year post-index period were significantly increased compared to the matched control groups (28.9% for the CKD3 patients and 111.3% for the CKD4), indicating the high economic burden of SHPT as well as a potential decreased quality of life due to these clinical events. Our findings are consistent with previously published literature. Chiroli et al. (2012) studied hemodialysis patients in 10 European countries in terms of healthcare resource use and costs. Similar to our findings, their analysis revealed that higher PTH levels resulted in increased hospitalization rates as well as significantly higher healthcare costs [24]. However, due to the different CKD stages at baseline, the results are not directly comparable to our findings. Schumock et al. (2008) assessed healthcare resource use and healthcare costs, as well as progression to dialysis in adult pre-dialysis CKD patients with and without SHPT using a large US administrative claims database [25]. They also found significantly higher resource utilization in the inpatient and outpatient sector, as well as significantly increased healthcare costs in all studied healthcare domains.

The disease progression to stage 5 in the present study was more than 5 times higher among CKD3 patients with SHPT and more than 9 times higher in CKD4 patients with SHPT, when compared to non-SHPT patients in the same baseline disease stage. This resulted in substantial differences in dialysis initiation as well. In line with these findings, Schumock et al. (2008) used Cox proportional hazards models to demonstrate that patients with CKD and SHPT were 4–5 times as likely to initiate dialysis when compared to patients with CKD and no SHPT claim [25].

Our results need to be interpreted considering several limitations, especially with respect to the used data source. Claims data are collected for the purpose of accounting and no clinical data are available. In this context, the unavailability of the glomerular filtration rate to assess CKD stage, as well as no clinical information on PTH levels to identify SHPT should be considered when interpreting the results, as the current analysis relied on the respective ICD-10-GM coding for CKD stages and SHPT, but also for other comorbidities such as renal osteodystrophy. The private medical insurance sector is not covered by this analysis, as the data pool only includes sickness funds from the SHI system only. However, only 11% of the German population is insured in the private sector. There is no ICD-10-GM code to identify specifically SHPT of renal origin in Germany, which is why we had to rely on the group code for “other diseases resulting from damage to the tubular kidney function”, which includes secondary hyperparathyroidism of renal origin but may also enclose patients with renal tubular acidosis. Given the low prevalence of renal acidosis, the potential bias from this inaccuracy can be neglected. Furthermore, as described above, SHPT patients were also identified by prescriptions for paricalcitol, alfacalcidol and calcitriol, while other vitamin D supplements were not considered as an indicator as they are often prescribed by general practitioners for other diseases or deficiencies. In line with this approach, patients were excluded from the non-SHPT cohorts if they presented with any paricalcitol, alfacalcidol and calcitriol prescriptions throughout the complete study period. However, comorbidities that might be treated with these agents were compared leading to a potential bias. Nonetheless, our goal was to keep the study populations as distinguished as possible. This study did not aim to create evidence on the epidemiology (incidence/prevalence) of SHPT. Hence, no assumption on epidemiological measures for SHPT in Germany can be drawn from our analysis. As this study did not intend to create epidemiological insights but to create a study population suitable for the assessment of the economic burden related to SHPT in CKD patients, the specific selection of our study population was considered a fair approach. Further, the results should be considered in line with the anticipated coding quality and diagnoses in the outpatient setting are coded on a quarterly basis and not attributed to an exact date.

Furthermore, as the analysis has shown, SHPT is generally an underdiagnosed condition, which is why it is likely that the non-SHPT cohort also included undiagnosed SHPT patients.

Lastly, due to the higher mortality in the control cohorts, average follow-up time was higher among SHPT patients which might result in higher healthcare resource use among these patients. However, healthcare resource use and healthcare spending generally tend to increase in the last months of life, and that effect was potentially stronger in the control cohorts, where more patients deceased. Despite these limitations, potentially giving rise to some extent of selection and classification bias, we believe that our study has important implications for the management of SHPT in Germany.

## Conclusions

Patients with CKD3 and 4 and incident SHPT of renal origin presented with significantly higher healthcare resource utilization and healthcare costs as well as disease progression to CKD5 and dialysis and had a higher prevalence of CVD compared to patients without SHPT during a 2-year follow-up period. These findings highlight the need to increase awareness of SHPT, as well as the need for early detection and new treatment options to mitigate the burden of disease for SHPT patients and on the healthcare system to possibly offset costs.

## Data Availability

The data used in this study was retrieved from the Institute for Applied Health Research Berlin (InGef) Research Database (https://www.ingef.de) and cannot be made available in the manuscript, the supplemental files, or in a public repository due to German data protection laws (Bundesdatenschutzgesetz). To facilitate the replication of results, anonymized data used for this study are stored on a secure drive at the Institute for Applied Health Research Berlin (InGef) GmbH. Access to the data used in this study can only be provided to external parties under the conditions of the cooperation contract of this research project and can be assessed upon request, after written approval at InGef GmbH.
